# Disassociated and concurrent structural and functional abnormalities in the drug-naïve first-episode early onset schizophrenia

**DOI:** 10.1007/s11682-021-00608-3

**Published:** 2022-02-18

**Authors:** Qiang Li, Sha Liu, Xiaohua Cao, Zexuan Li, Yun-shuang Fan, Yanfang Wang, Jiaojian Wang, Yong Xu

**Affiliations:** 1 Shanxi Key Laboratory of Artificial Intelligence Assisted Diagnosis and Treatment for Mental Disorder/Department of Psychiatry, First Hospital of Shanxi Medical University, No. 85 Jiefang Nan Road, Taiyuan, China; 2 Department of Psychiatry, First Hospital/First Clinical Medical College of Shanxi Medical University, Taiyuan, China; 3 School of Life Science and Technology, University of Electronic Science and Technology of China, Chengdu, 625014 China; 4 Center for Language and Brain, Shenzhen Institute of Neuroscience, Shenzhen, 518057 China

**Keywords:** Early onset schizophrenia, Functional connectivity density, Grey matter volume, Left lateral orbitofrontal cortex

## Abstract

Schizophrenia which is an abnormally developmental disease has been widely reported to show abnormal brain structure and function. Enhanced functional integration is a predominant neural marker for brain mature. Abnormal development of structure and functional integration may be a biomarker for early diagnosis of schizophrenia. Fifty-five patients with early onset schizophrenia (EOS) and 79 healthy controls were enrolled in this study. Voxel-based morphometry (VBM) and functional connectivity density (FCD) were performed to explore gray matter volume (GMV) lesion, abnormal functional integration, and concurrent structural and functional abnormalities in the brain. Furthermore, the relationships between abnormalities structural and function and clinical characteristics were evaluated in EOS. Compared with healthy controls, EOS showed significantly decreased GMV in the bilateral OFC, frontal, temporal, occipital, parietal and limbic system. EOS also showed decreased FCD in precuneus and increased FCD in cerebellum. Moreover, we found concurrent changes of structure and function in left lateral orbitofrontal cortex (lOFC). Finally, correlation analyses did not find significant correlation between abnormal neural measurements and clinical characteristic in EOS. The results reveal disassociated and bound structural and functional abnormalities patterns in EOS suggesting structural and functional measurements play different roles in delineating the abnormal patterns of EOS. The concurrent structural and functional changes in lOFC may be a biomarker for early diagnosis of schizophrenia. Our findings will deepen our understanding of the pathophysiological mechanisms in EOS.

**Supplementary Information:**

The online version contains supplementary material available at 10.1007/s11682-021-00608-3.

## Introduction

Early onset schizophrenia (EOS) defined as schizophrenia beginning before the 18 years of age is more severe and less effected by environment and medication compared with late onset schizophrenia, which provides unique opportunity to explore pathophysiological mechanisms of schizophrenia (Kyriakopoulos et al., 2008; Yang et al., 2014). Recently, abnormal brain structural alterations have been widely reported in EOS (Douaud et al., 2009; Epstein et al., 2014). Significantly reduced gray matter volume (GMV) in EOS, and even in ultra-high risk of psychosis individuals has been reported, although the reduction in the latter is less obvious than that in schizophrenia (Douaud et al., 2007; Nenadic et al., 2015; Thompson et al., 2001). Reduced GMV in the high-order cortical areas were positively correlated to cognitive impairments and negatively correlated with psychiatric symptoms such as hallucination in schizophrenia (Kanahara et al., 2013; Kubota et al., 2015; Ota et al., 2017; Qiu et al., 2018). Therefore, the GMV abnormality might contribute to the underlying pathogenic mechanism of schizophrenia. A large number of previous studies have demonstrated that structure determines functions (Fan et al., 2020; Honey et al., 2009; J. Wang et al., 2019a, 2019b; Wang et al., 2017a; Wang et al., 2015; Wang et al., 2016). Thus, to reveal the concurrent structural and functional changes, especially in functional integration may be an early biomarker for early diagnosis of EOS.

Schizophrenia has been considered to be a developmental psychiatric disorder manifesting abnormal functional integration. To characterize the functional integration of brain, functional connectivity density (FCD) was developed and has been widely used to study the abnormal functional integration in brain related disorders (C. Liu et al., 2018; Wang et al., 2017b). Recently, FCD has also been applied to study abnormal functional integration in adult schizophrenia and observed significantly increased FCD in the striatum, hippocampus, default mode network (DMN), thalamus, temporal and decreased FCD in the sensorimotor cortex, temporal-occipital conjunction and calcarine sulcus (Huang et al., 2017; Chuanxin Liu et al., 2017; Zhuo et al., 2014). All these studies suggested that FCD is a reliable approach to characterize functional integration and can effectively delineate the underlying neuropathology of schizophrenia. Although abnormal functional integration has been identified in adult schizophrenia, how the abnormal development of functional integration contributes to the onset of EOS remains unclear.

Based on previous findings in adult schizophrenia, we hypothesized that GMV and FCD exhibited different and concurrent changing patterns in EOS compared to healthy controls. To reveal different and concurrent abnormal structural and functional patterns in EOS, 55 drug-naïve EOS and 79 healthy controls were included in this study. First, GVM and FCD were separately applied to reveal the changed structural and functional integration patterns. Next, the brain areas with concurrently abnormal GMV and FCD were identified. Finally, the correlation analyze were preformed to reveal the associations between abnormal neural indices and clinical characteristics.

## Material and methods

### Participants

A total of 55 patients with EOS (35 females and 20 males, age range 9.0–17.9 years, mean ± SD = 14.9 ± 1.5 years) were recruited from the Department of Psychiatry at the First Hospital of Shanxi Medical University, Shanxi China. Seventy-nine age and gender matched healthy controls (HCs) (46 females and 33 males, age range 7.5–17.9 years, mean ± SD = 14.3 ± 2.2 years) were enrolled from local community through advertisements. All patients were assessed by two well-trained psychiatrists based on the Structured Clinical Interview for DSM-IV-TR, patient version (SCID-I/P). The inclusion criterial were as the follows: (1) no-morbid Aix-I or Axis-II diagnose; (2) duration of illness less than 1 year. In addition, all patients were interviewed 6 months later again to confirm a final diagnose of schizophrenia. Clinical symptoms in patients were accessed using the Positive and Negative Syndrome Scale (PANSS). All patients were in their first episode and were drug-naïve before the neuroimaging scanning. All the patients and HCs with substance use disorder, any past or current neurological disorder or first-degree relatives history of hereditary neurological disorders, history of head injury with loss of consciousness, pregnancy, other comorbid mental disorders, and MRI-contraindications were excluded in current study. All participants informed written consents were given and obtained. This study was approved by the Ethics Committee of the First Hospital of Shanxi Medical University.

### MRI data acquisition and preprocessing

MRI scanning was performed by using a Siemens Trio 3.0 Tesla scanner (Erlangen, Germany). Participants were instructed to stay awake with their eyes closed during the scan. Functional images were acquired using an echo-planar imaging (EPI) sequence with the following parameters: repetition time (TR) = 2500 m s; echo time (TE) = 30 m s; matrix = 64 × 64, 32 axial slices; slice thickness = 3 mm with 1 mm gap; flip angle = 90°; field of view = 240 × 240 mm^2^; voxel size = 3.75 × 3.75 × 4 mm^3^; and 212 volumes. T1-weighted anatomical images were acquired as a three-dimensional fast-spoiled gradient-echo sequence with the following parameters: TR = 2300 m s; TE = 2.95 m s; matrix = 240 × 256, 160 axial slices; slice thickness = 1.2 mm, no gap; flip angle = 9°; field of view = 225 × 240 mm^2^; and voxel size = 0.9375 × 0.9375 × 1.2 mm^3^.

Rest-state functional MRI data preprocessing was carried out by using DAPBI (http://rfmri.org/dpabi) software toolbox. The first 10 functional volumes were discarded to ensure for equilibration of magnetic field. Next, slice timing and realignment correction were performed to correct temporal difference between slices and head motion between time points. Twelve EOS patients and 11 HCs were excluded due to head motion exceeding 2.0 mm or 2° in any direction, and finally 55 patients and 79 HCs were used to explore the association between FCD and GMV. Furthermore, all corrected images were normalized into standard stereotactic EPI temple in Montreal Neurological Institute (MNI) space and resampled to voxel size of 3 × 3 × 3mm^3^. The normalized images were linearly detrended and nuisance covariates including Fristion 24 motion parameters (Friston et al., 1996), WM signal, CSF signal and whole-brain global signal were regressed out by the multiple regression model to reduce effects of head motion and non-neuronal blood oxygenation levels-dependent (BLOD) fluctuations (Tomasi & Volkow, 2012). Finally, all fMRI imaging time-series underwent band-pass temporal filtering (0.01–0.08 Hz). In addition, the mean frame-wise displacement (FD) was calculated and fed to statistical analyses to further remove the effects of head motion (Power et al., 2012; L. Wang, Yu, Wu, Wu, & Wang, 2019).

### Voxel-based morphology (VBM) analysis

VBM method was used to investigate changes of GMV in EOS. High spatial resolution T1 weighted MR images of all subjects were processed using the Computational Anatomy Toolbox (CAT12; http://dbm.neuro.uni-jena.de/cat/) implemented in SPM12 (http://www.fifil.ion.ucl.ac.uk/spm/software/spm12/). All T1-weighted MR images origins were first manually corrected to anterior–posterior commissure. Then, all the corrected T1 images were segmented into gray matter (GM), white matter (WM) and cerebrospinal fluid (CSF) using standard unified parameters and adjusted by modulation. After segmentation, the GM map was registered into the MNI space using both linear and nonlinear affined transformation to a voxel size of 1.5 × 1.5 × 1.5mm^3^. We also calculated total intracranial volume (TIV) for all subjects as covariate regressed during statistical analysis to correct for individual brain size and volume effects. Finally, all GM images were smoothed using a 6-mm FWHM Gaussian Kernel for statistics.

### FCD analysis

The FCD mapping was calculated by using Dynamic BC toolbox (Liao et al., 2014). Based on the processed data, the FCD calculation was constrained to a whole brain mask. Global FCD (gFCD) was used to characterize the functional integration in this study. gFCD was defined as number of voxels showing functional connectivities with whole brain above a given correlation coefficient threshold. Here, the correlation coefficient threshold was set at *R* > *0.6* which was demonstrated to be able to effectively reduce false positive rates and is higher sensitive and most stable than other thresholds to identify the functional module of the brain (Tomasi et al., 2016). The FCD map of each participant then was rescaled thought calculating the standard deviation across whole brain voxels to reduce effect of individual variability and improve normality. Finally, the FCD map was obtained for each subject and was smoothed with a 8 mm isotropic Gaussian kernel for statistical analyses (Tomasi & Volkow, 2011).

### Statistical analyses

Two-sample t-tests were performed to investigate GMV and FCD group differences, age, gender, TIV (GMV) and mean FD (FCD) were regressed out as covariates. To reveal the concurrent changes of GMV and FCD, regions showed abnormal GMV or FCD were taken as regions of interest (ROIs), and FCD or GMV values were extracted from these ROIs (Table 1). Two sample t-tests with age, gender and regional average GMV as covariates were used to explore whether the FCD or GMV in these ROIs, were significant different between patients and controls. Moreover, partial correlation coefficients were calculated to quantify the relationship between regional FCD and GMV alterations. To further validate the results, we further compared these values between groups by not regressing out regional GMV.

**Table 1:** Local peaks of the brain areas showed reduced GMV in the patients group compared with healthy controls (HCs) (*FDR* corrected, *p*<0.05)

Regions	L/R	Abbeviation	MNI (mm)			t-value
			x	y	z	
Angular gyrus	R	AG	27	-59	44	3.36
Cerebellum_6	R	Cereb	33	-60	-24	4.69
Cerebellum anterior lobe		aCereb	8	-65	-9	3.19
Verimis_9		Ver9	-3	-54	-30	4.04
Middle cingulate cortex	R	MCC	11	35	30	4.46
Lateral orbitofrontal cortex	L	lOFC	-26	32	-14	4.00
Lateral orbitofrontal cortex	R	lOFC	21	14	-22	4.58
Superior frontal gyrus	L	SFG	-24	-9	57	5.14
Medial prefrontal gyrus	R	MPFC	-42	14	39	3.73
Fusiform gyrus	L	FG	-21	-71	-17	4.26
Hippocampus	L	Hipp	-15	-6	-14	4.00
Insula	L	INS	-36	14	9	4.09
Middle occipital gyrus	L	MOG	-27	-85	29	3.38
Paracentral gyrus	R	PG	14	-30	54	2.75
Superior parietal gyrus	L	SPG	-24	-72	51	3.25
Inferior parietal gyrus	L	IPG	-32	-74	39	4.17
Postcentral gyrus	L	ParaG	-44	-35	47	3.46
Postcentral gyrus	R	POG	-35	-27	48	4.24
Inferior temporal gyrus	L	ITG	-50	-22	-21	4.10
Middle temporal gyrus	L	MTG	-65	-17	-14	4.88
Superior temporal gyrus	L	STG	-54	-8	6	3.55
Superior temporal gyrus	R	STG	63	-32	2	3.63

Abbreviations: GMV, gray matter volume; L, left side; R, right side;

### Correlation analyses

Spearman’s correlation analyses were used to test the relationships between neuroimaging measurement and clinical features such as PANSS. Age, gender was considered as covariates to exclude the confounding effects. False Discovery Rate (FDR) method with *p* < 0.05 was used for multiple comparison correction.

## Results

### Participants and demographic characteristics

The demographic and clinical features of all subjects are listed in Table 2. No significant difference in age (Mann–Whitney *U* test, *p* = 0.28) and gender (*χ*^*2*^ = 0.40, *p* = 0.53) were found between EOS and HCs. We also did not find the significant differences in whole brain GMV (*p* = 0.071) and mean FD (*p* = 0.17) between EOS and HCs.

**Table 2:** Demographics data of early-onset schizophrenic (EOS) and healthy controls (HCs)

Characteristic	EOS	HC	value
	Mean ± SD	Mean ± SD	
Sample size	55	79	
Age (years)	14.9±1.53	14.3±2.17	0.2816^a^
Gender(F/M)	35/20	46/33	0.5288^b^
handedness	55\0	79\0	
TIV	1438.8±128.7	1398.5±127.2	0.0714^c^
Mean FD	0.144±0.10	0.15±0.08	0.1725^a^
Total score (PANSS)	65.4±18.3		
Positive score	14.9±5.16		
Negative score	13.9±5.88		
General	31.7±8.95		

Abbreviations: FD, frame-wise displacement; TIV, total intracranial volume; PANSS, Positive and Negative Syndrome Scale; Mean ± SD, values are mean ± standard deviation; F/M, Female/Male

^a^ Mann–Whitney U-test

^b^ Chi-square test

^c^ Unpaired t test

### GMV alterations in patients with EOS

**Fig.1:**
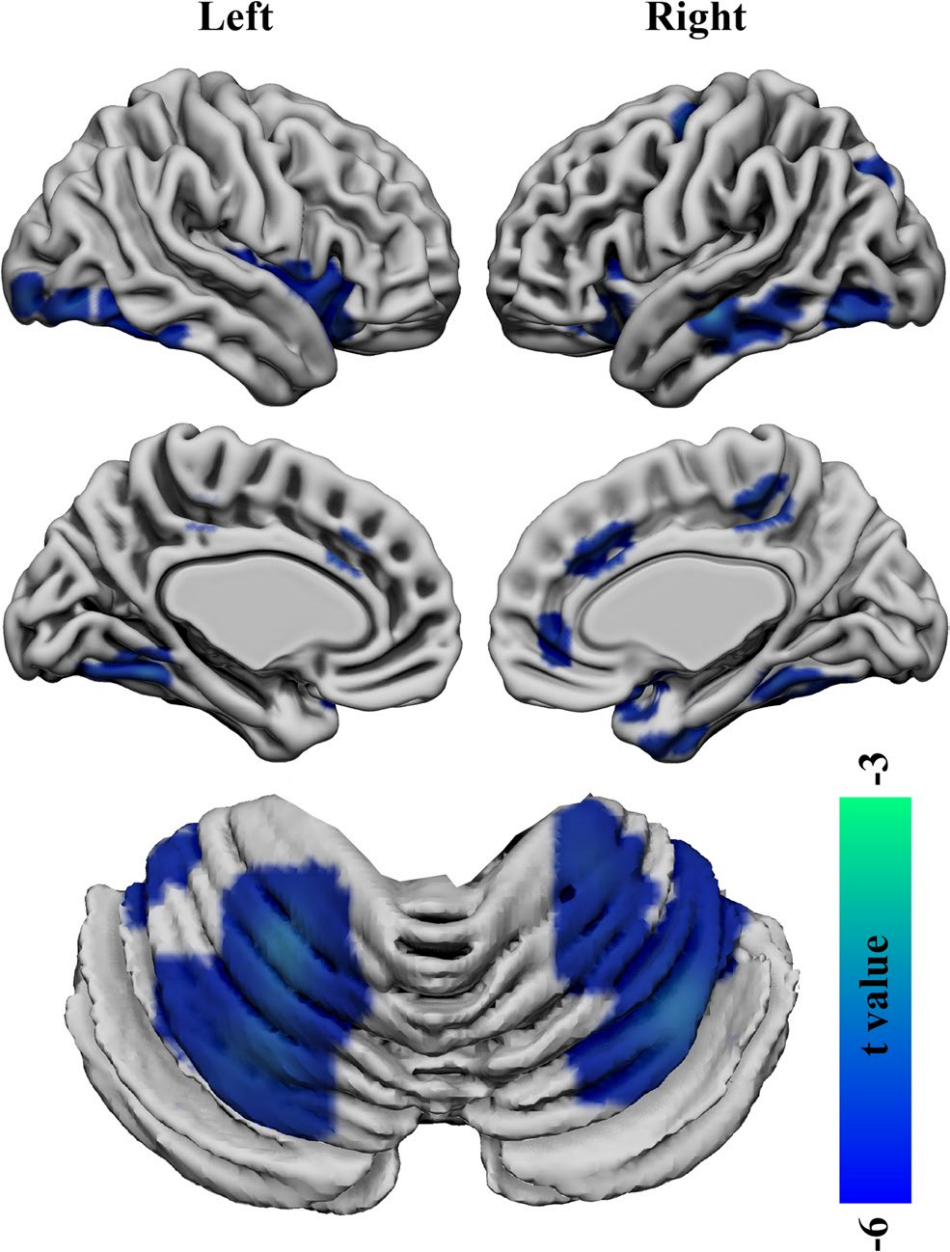
**Decreased grey matter volume (GMV) in the drug-naïve first-episode early-onset schizophrenia (EOS) compared with healthy controls (HCs).** Regions (colors of blue and green) showed significantly reduced GMV in the bilateral cerebellum anterior–posterior lobes, temporal, orbitofrontal, occipital, parietal, limbic lobes, sensorimotor areas, cingulate cortex and the precuneus in EOS compared with HCs (*FDR* corrected, *p* < 0.05; minimum cluster size of 50 voxels)

Compared with HCs, significantly reduced GMV were found in the bilateral cerebellum, temporal, orbitofrontal cortex (OFC), occipital, parietal, limbic lobes, hippocampus, insula, sensorimotor areas, cingulate cortex and precuneus in EOS (*p* < 0.05, *FDR* corrected; minimum cluster size of 50 voxels, Fig. 1). No significantly increased GMV has been found in EOS.

### FCD alterations and its relationships with GMV

**Fig.2:**
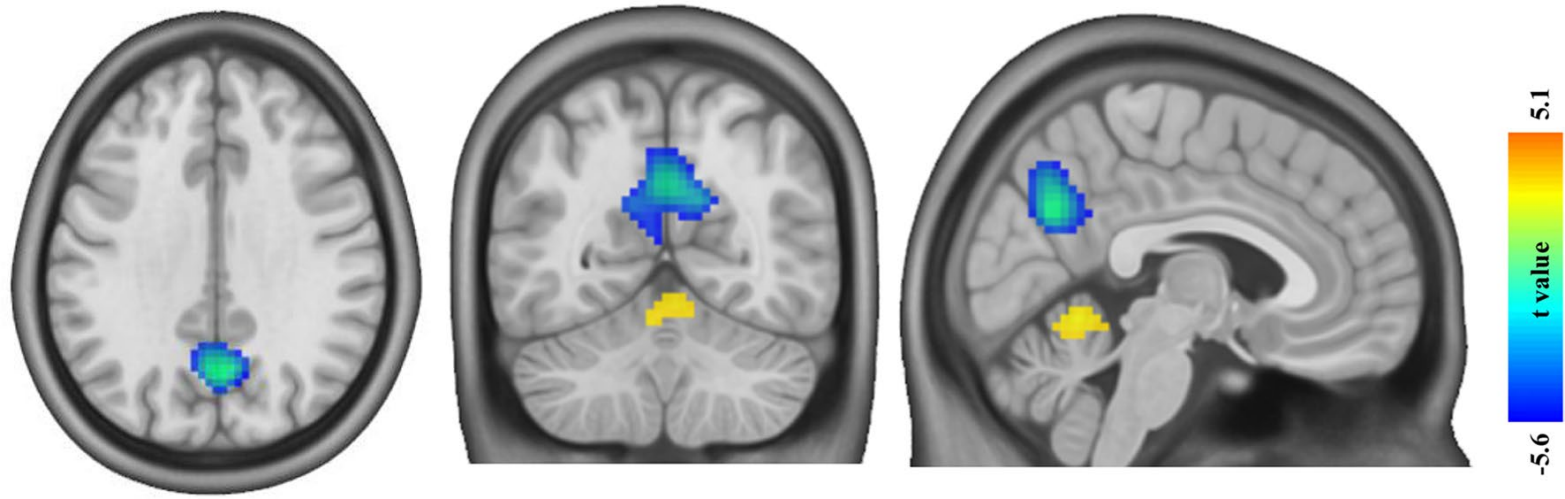
**The regions of altered functional connectivity density (FCD) in early-onset schizophrenia (EOS) compared with healthy controls (HCs).** EOS showed increased FCD in the bilateral cerebellum vermis (warm color) and decreased FCD in the bilateral precuneus compared to HCs (winter color) (*FDR* corrected, *p* < 0.05; minimum cluster size of 100 voxels)

**Fig.3:**
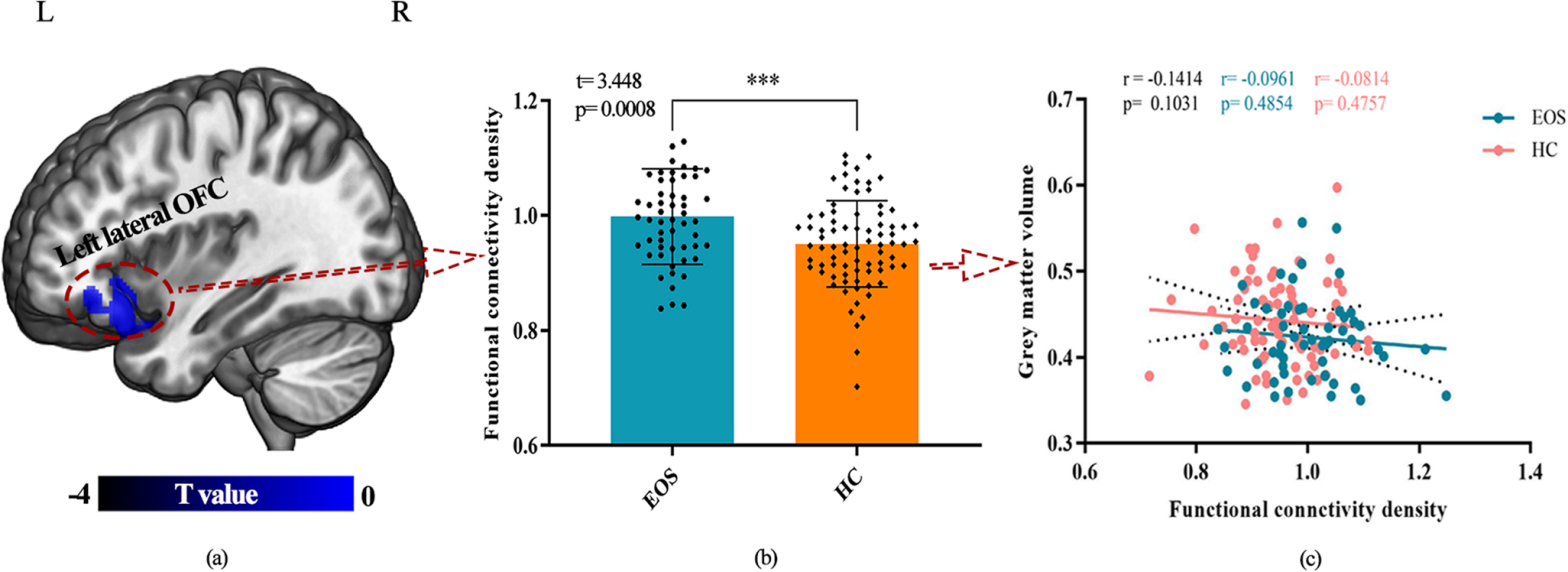
**Concurrent changes of GMV and FCD and their association in the left orbitofrontal cortex (OFC).** The left OFC (MNI: -26, 32, -14) showed significantly decreased GMV while increased FCD in EOS compared with controls: Left panel, the left lateral orbitofrontal cortex (lOFC) showed significantly decreased GMV in patient. Middle panel, significantly increased FCD in the left lOFC was identified with age, gender and GMV as covariances. Right panel, there is no significant correlation between increased FCD and decreased GMV in left lOFC. *** *p*<0.001

Using two-sample t-test, patients with EOS showed significantly increased FCD in the cerebellum vermis and decreased FCD in the precuneus (*p* < 0.05, *FDR* corrected; minimum cluster size of 100 voxels, Fig. 2). In these regions, no significant differences in GMV were found in patients with EOS compared to HCs (Supplementary Figure S2). Taking regions with reduced GMV as ROIs, EOS patients showed significantly increased FCD in the left OFC after regressing out the effects of age, gender and GMV (Fig. 3). The correlation analysis between increased FCD and decreased GMV in the left OFC did not find significant correlation suggesting asynchronized changes of GMV and FCD in this area (Fig. 3). Additionally, the left OFC also showed increased FCD in EOS when only regressing out age and gender not including regional GMV (Supplementary Figure S3).

### Relationships between neuroimaging measurement and clinical variables

No significant correlation was found between neuroimaging indices and PANSS score in EOS patients (*p* > 0.05; Supplementary Figure S4).

## Discussion

In our study, we identified diffusely reduced GMV in the bilateral OFC, sensorimotor areas, high-order network cortex, visual cortex and limbic system in EOS patients. We also found significantly increased FCD in the cerebellum and decreased FCD in the precuneus, but GMV differences in these regions were not observed. Moreover, we found concurrent changes of GMV and FCD in left lateral OFC in EOS. Our findings may provide the neuroanatomical basis for the onset of EOS and highlighted the important role of left lateral OFC which may serve as an early biomarker for diagnosis of EOS.

In our study, regions showing significantly decreased GMV were consistent with the findings reported in previous studies (Douaud et al., 2007; Gogtay et al., 2007; Rapoport & Gogtay, 2011). The GMV deficits in schizophrenia at an early age may reflect an illness process and abnormal neurodevelopment, which was closely related to cognitive impairments in working memory and language found in EOS (Lieberman et al., 2005; Pantelis et al., 2003; White et al., 2006). These brain structural deficits at early stage may contribute to the onset and progression of EOS (Thompson et al., 2001).

In addition, EOS patients showed decreased FCD in precuneus and increased FCD in cerebellum. Our findings were supported by previous studies that also reported aberrant functional integration capacity in precuneus and cerebellum in adult schizophrenia (Huang et al., 2017; Zhuo et al., 2018). The precuneus is a key node of the default mode network (DMN) and plays an important role in self-reflection processing, autobiographical memory retrieval and envisioning future events (Buckner et al., 2008; J. Wang et al., 2019a, 2019b). The disrupted functional integration of precuneus may be related to cognitive impairments and pathogenesis of schizophrenia (Hu et al., 2017). Cerebellum connected to cortical and subcortical regions forming cortico-cerebellar-thalamic-cortical circuit (CCTCC). The disrupted functional integration of cerebellum could affect the whole systems and lead to diversity symptoms and social cognitive deficits in schizophrenia (Adamaszek et al., 2017; Andreasen & Pierson, 2008). Our current results indicated that drug-naïve EOS patients have showed aberrant functional connectivity patterns in DMN and CCTCC at early stage of disease.

Notably, both the structure and function of the left lateral OFC were affected by the illness in EOS patients. OFC which was found to receives sensory stimuli information was associated with decision making, reward processing and inhibition control from other key regions such as amygdala, striatum, thalamus and hippocampus (Bechara et al., 2000; Jennings et al., 2019; Sakurai et al., 2015). OFC is also critical for maintaining normal social interconnection (Rudebeck & Rich, 2018). The previous studies found that patients with schizophrenia exhibited decreased GMV in the OFC associated with altered attention mode, poor recognition performance (Herold et al., 2009; Karatekin & Asarnow, 1999) and cognitive impairments as core features for schizophrenia (Kahn & Keefe, 2013). Furthermore, reduced GMV of OFC was significantly correlated to hallucination severity in schizophrenia (Qiu et al., 2018). Moreover, as a crucial node of the high order cognitive network (Tomasi et al., 2016), dysfunction of OFC and its functional integration is highly related to cognitive, emotion, behavior and clinical features in psychiatric disorders and FCD value in OFC can effectively classify the schizophrenia from depression (Chen et al., 2017; Rudebeck & Rich, 2018). Increased FCD in EOS found in our study was supported by previous studies which showed hyper-connectivity of OFC in schizophrenia, especially at the early stage of schizophrenia. And the hyper-connectivity of OFC may be a compensatory for other functionally abnormal brain areas or as compensatory reaction for anatomical impairments (Fornito et al., 2012, 2017). Some recent studies found that disruption of left inferior frontal-occipital fasciculus and left inferior longitudinal fasciculus which connected with left OFC contributes to high-order cognitive impairments in EOS (Epstein et al., 2014; Liu et al., 2014). Therefore, aberrant functional integration capacity of OFC might be a compensatory reaction to maintaining normal brain function in early onset of illness. Above all, our findings suggested that structural and functional alterations in left OFC may be a risk factor for development of EOS.

To delineate the order of structural and functional changes during disease is a fundamental question in clinical studies. Recent study in schizophrenia revealed structural and functional segregation in left OFC. Xu et al. found that abnormal FC patterns in the OFC were not influenced by GVM lesions in schizophrenia (Xu et al., 2017). A similar research also found that altered FCD in cortical, sub-cortical regions and limbic system were not affected by GMV reduced in schizophrenia (Zhuo et al., 2017). There are two main theories to explain the disassociation between GMV and FCD in OFC. One is that the disassociation between FCD and GMV reflected strong robustness and resilience to cope with focal neural injury as potential functional advantage in brain region (Fornito et al., 2012; Lynall et al., 2010). The other is that dysfunctional integration of high-order brain regions is affected by anatomical connection, rather than focal neural lesions (van den Heuvel & Fornito, 2014). Therefore, that structural and functional connectivity abnormalities may be contribute independently to the pathophysiology of EOS. Interestingly, although we found concurrent changes of GMV and FCD in left lateral OFC, we did not find the significant association between GMV and FCD in this area. This finding suggested that structural and functional changes did not simultaneously occur in the OFC. Given that the areas with changed FCD did not show the structural changes but structurally abnormal brain areas showing FCD differences, our findings suggested that the functional abnormalities may occur before structural abnormalities in left OFC in EOS.

In current study, we did not find any significant associations between alterations of FCD/GMV and PANSS scores in EOS group, suggesting that the altered functional integration and structural lesions of left lateral OFC were independently of the severity of clinical symptoms. Previous studies have demonstrated that severity symptoms such as hallucination might be associated with right OFC (Qiu et al., 2018) and altered FCD in the DMN was negativity correlated with the scores of PANSS in EOS (X. Wang et al., 2017c). Several limitations of the current study should be considered. First, the current study lacked relevant assessments, such as cognitive function, behavior states, motion function and intelligence quotient (IQ) tests, this information will be collected to investigate the relationships between neuroimaging and disease features in EOS. Second, our study needs to further explore correlations between functional integration patterns and anatomical connections may further enhance our understanding of the potential pathological mechanism of EOS. Third, a smaller samples size would result in unstable statistical results, larger samples will be necessary in future study. Finally, registering EOS patient brains to the MNI standard template could result in some age-specific differences in spatial normalization. However, these differences are unlikely to affect the fMRI results because fMRI has relatively lower spatial resolution (Alexander-Bloch et al., 2010; Burgund et al., 2002; Kang et al., 2003). It is merited to apply participant-specific brain templates when performing registration steps in further research.

## Conclusions

Our study revealed distributed structure abnormalities in temporal, frontal, parietal, and subcortical areas and abnormal functional integration in precuneus and cerebellum. We also identified concurrent changes of structural and functional integration in left OFC. Our findings revealed dissociated and bounding structural and functional abnormalities patterns in drug-naive EOS. The concurrent structural and functional changes in OFC demonstrated a potential functional advantage of OFC which has strong resilience to cope with focal neural injury and increased FCD in the OFC may represent a compensatory processing to maintaining normal brain function in EOS.

## Supplementary Information

Below is the link to the electronic supplementary material.

Supplementary file1 (DOC 923 KB)
