# Supplementary material for: Disassociated and concurrent structural and functional abnormalities in the drug-naïve first-episode early onset schizophrenia
Source: Brain Imaging Behav. 2022 Feb 18;16(4):1627–35. doi: 10.1007/s11682-021-00608-3 (PMC9279212; doi:10.1007/s11682-021-00608-3)
Supplement: Supplementary file 1 — Supplementary file1 (DOC 923 KB) [file 11682_2021_608_MOESM1_ESM.doc]

Supplementary 1

**
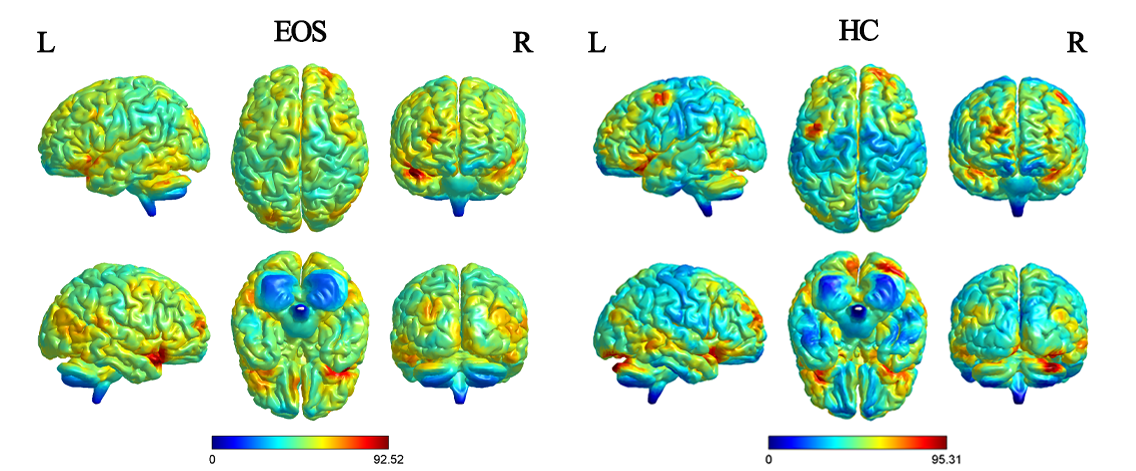
**

**Figure.S1 The FCD distribution pattern in early onset schizophrenia (EOS) and healthy controls (HCs).** One sample t tests were used to map the distribution patterns of FCD in both EOS and HCs (*FDR* corrected, *p*﹤0.05).

**
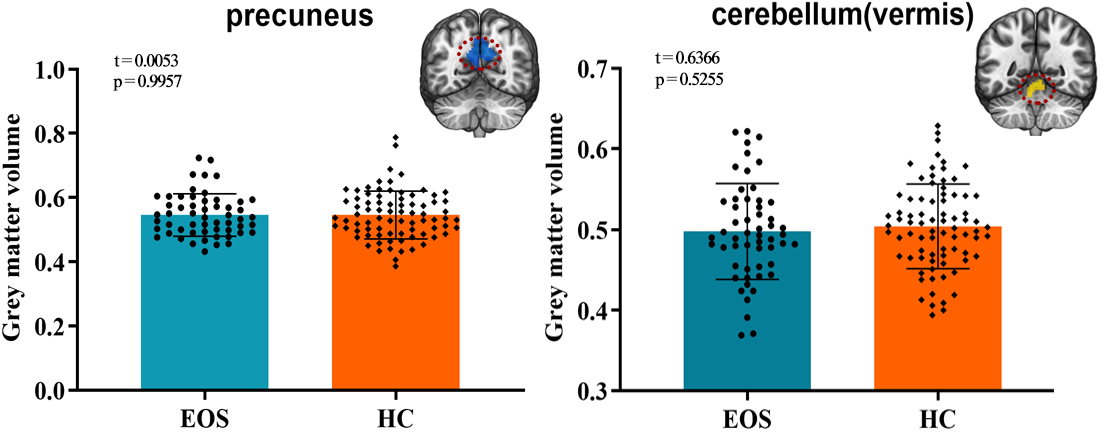
**

**Figure.S2 GMV in the regions with changed FCD.** The mean GMV in the brain areas with changed FCD was extracted and compared between EOS and HCs. No significant differences were found between EOS and HCs.


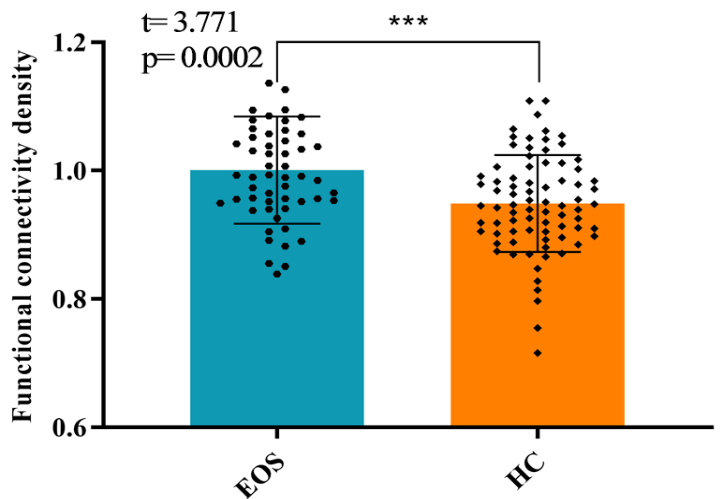


**Figure.S3 Altered FCD in the left orbitofrontal cortex (OFC) without correction of GMV in EOS**. The left OFC showed significantly increased FCD in EOS compared with controls: without correction for GMV. *** *p*﹤0.001


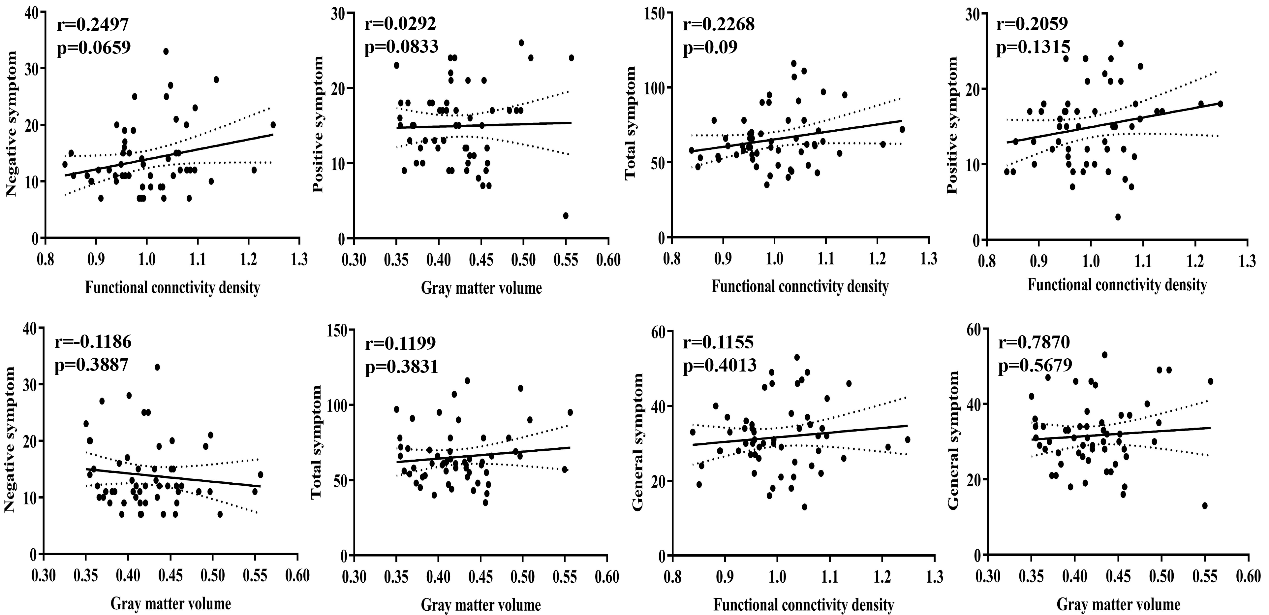


**Figure.S4** **Correlation analyses between decreased GMV, increased FCD and PANSS scores.** No significant correlations were found between alterations of FCD or GMV and clinical features (PANSS) in EOS (*Ρ* >0.05).

**
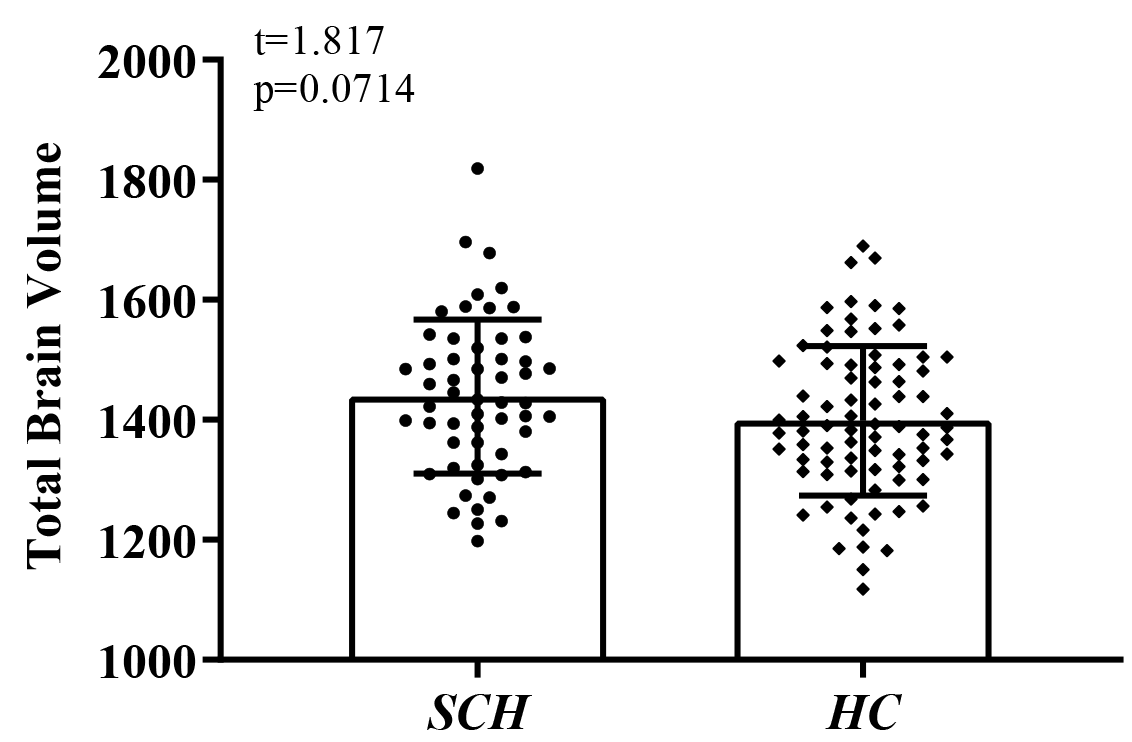
**

**Figure.S5 Group differences between EOS and HCs in total brain volume.** There were no significant group differences between EOS and HCs in total brain volume ( *t* = 1.817, *p* = 0.0714).
